# Explanation of the personality factor with the Enneagram in the selection of the specialty branch of the intern doctors

**DOI:** 10.1192/j.eurpsy.2024.1365

**Published:** 2024-08-27

**Authors:** B. Özen, E. Ilgin, E. Akça, A. Özercan, P. Seçgin, Ö. Yanartaş

**Affiliations:** Psychiatry, Marmara University Research & Training Hospital, Istanbul, Türkiye

## Abstract

**Introduction:**

Personality characteristics have an important place in the choices of interns who are at the stage of deciding how their profession will be shaped in the future. While the Big 5 personality model has been widely used in evaluating the personality traits factor in career planning, the Enneagram has increased in popularity in recent years. In this study, it was aimed to investigate how senior medical students evaluate their professional future between these choices and the students’ personality types.

**Objectives:**

Forms and scales were presented to 221 interns who agreed to participate in our study and were studying in their final year in the 2022-2023 period at three different faculties, two state universities and one private university in the Marmara Region in Turkey.

**Methods:**

The sociodemographic data form, Enneagram Personality Types and Subtypes Inventory, and Positive Future Expectation Scale, prepared by the researchers and containing questions about the factors that may be effective in choosing medical specialization, obtained through a face-to-face pilot interview with ten students and literature review, were applied to the participants. Participants answered the forms and scales via ‘Google forms’.

**Results:**

211 out of 221 participants, who did not constitute outliers, were included in the analysis. The mean age of the participants was 24.43 (S.E= 0.11)

In terms of Enneagram typologies, Type 2 (39.3%) exhibited the highest prevalence, followed by Type 1 (13.3%), Type 6 (11.8%), and Type 7 (8.5%).

Furthermore, a statistically significant relationship was found between specialization area and Enneagram types (Fisher exact <.001, p< .001). Post-hoc examinations highlighted specific associations, such as the relationship between Type 3 and Cardiovascular Surgery, Orthopedics and Traumatology; Type 4 and Pneumology, Psychiatry; Type 5 and PRC, Type 6 and Infectious Diseases, Neurology, Medical Microbiology; Type 7 and Cardiology; Type 8 and Pediatrics, Medical Biochemistry; and Type 9 and Family Medicine, Radiology, Psychiatry, Medical Pathology.

**Conclusions:**

When the results are evaluated, the highest rate of type 2 and type 1 of the Enneagram typology in senior medical faculty students supports the fact that the medical profession consists of responsible and principled people who love helping others. It is compatible with the character traits of people with type 9 who avoid stress and conflict, preferring the department to have a low workload, and turning to family medicine, radiology, psychiatry and medical pathology departments, which are estimated to have relatively fewer working hours and emergency applications.

Our study suggests that this scale be used more widely, as the Enneagram typology, which is used in many professional and career choices, shows results compatible with the participants’ preferences in choosing a medical specialty.

**Disclosure of Interest:**

None Declared

